# Melatonin: An Anti-Tumor Agent in Hormone-Dependent Cancers

**DOI:** 10.1155/2018/3271948

**Published:** 2018-10-02

**Authors:** Javier Menéndez-Menéndez, Carlos Martínez-Campa

**Affiliations:** Department of Physiology and Pharmacology, School of Medicine, University of Cantabria and Instituto de Investigación Valdecilla (IDIVAL), 39011 Santander, Spain

## Abstract

Melatonin (N-acetyl-5-methoxytryptamine) is a hormone synthesized and secreted by the pineal gland mainly during the night, since light exposure suppresses its production. Initially, an implication of this indoleamine in malignant disease was described in endocrine-responsive breast cancer. Data from several clinical trials and multiple experimental studies performed both *in vivo* and *in vitro* have documented that the pineal hormone inhibits endocrine-dependent mammary tumors by interfering with the estrogen signaling-mediated transcription, therefore behaving as a selective estrogen receptor modulator (SERM). Additionally, melatonin regulates the production of estradiol through the control of the enzymes involved in its synthesis, acting as a selective estrogen enzyme modulator (SEEM). Many more mechanisms have been proposed during the past few years, including signaling triggered after activation of the membrane melatonin receptors MT-1 and MT-2, or else intracellular actions targeting molecules such as calmodulin, or binding intranuclear receptors. Similar results have been obtained in prostate (regulation of enzymes involved in androgen synthesis and modulation of androgen receptor levels and activity) and ovary cancer. Thus, tumor metabolism, gene expression, or epigenetic modifications are modulated, cell growth is impaired and angiogenesis and metastasis are inhibited. In the last decade, many more reports have demonstrated that melatonin is a promising adjuvant molecule with many potential beneficial consequences when included in chemotherapy or radiotherapy protocols designed to treat endocrine-responsive tumors. Therefore, in this state-of-the-art review, we aim to compile the knowledge about the oncostatic actions of the indoleamine in hormone-dependent tumors, and the latest findings concerning melatonin actions when administered in combination with radio- or chemotherapy in breast, prostate, and ovary cancers. As melatonin has no toxicity, it may be well deserve to be considered as an endogenously generated agent helpful in cancer prevention and treatment.

## 1. Introduction

It was in 1958 when Lerner et al. reported the isolation and identification of a substance from the pineal gland from beef [1]. This new molecule, a derivative of tryptophan, is an indoleamine (N-acetyl-5-methoxytryptamine) that was called melatonin, name derived from its effect in blanching the melanophores (mela-) in amphibians and because the precursor to melatonin is serotonin (-tonin).

Melatonin is a hormone mainly secreted during the night, and, since its discovery, many physiological functions have been attributed to this indoleamine, which is a pleiotropic molecule that acts as a hormone in mammals. We can highlight among its functions: (i) the control of the seasonal reproduction [2]. (ii) The ability to reduce the oxidative stress either by direct detoxification or indirectly (inhibiting the activity of prooxidative enzymes and stimulating antioxidant enzymes) [3]. (iii) The effect on the immune system by enhancing both natural and acquired immunity in mammals [4]. (iv) Its function as a circadian rhythm synchronizer of the sleep-wake cycle acting as a physiological sleep regulator [5]. (v) The ability to prevent cancer, an inhibitory effect involving both membrane receptor-dependent and independent mechanisms at the initiation, promotion, progression, and malignant metastasis phases [6–8].

More than eighty percent of the works trying to elucidate the implications of melatonin in cancer have been published in the recent two decades, which highlights the importance of this field of research. The role of melatonin has been extensively investigated in many different neoplasias, and there is growing evidence that the pineal hormone is associated with a lower risk of cancer as demonstrated in different *in vivo* and *in vitro* models [6, 9].

Most of the anticancer effects of melatonin were initially described on endocrine mammary tumor models. However, hundreds of recent reports demonstrate an anticancer effect of the pineal hormone on many other kinds of cancers. Therefore, the main objective of this review is to compile the state-of-the-art of the current knowledge about melatonin oncostatic effects on estrogen-dependent breast and ovarian cancers and in androgen-dependent prostate cancer. We will pay special attention to the intracellular signaling pathways that are usually altered after melatonin addition, particularly when it is coadministered either with conventional chemotherapeutic drugs or with radiotherapy.

## 2. Melatonin and Hormone-Dependent Breast Cancer

Breast development at puberty and during sexual maturity is stimulated by estradiol, which is the most physiologically active hormone in breast tissues. However, estrogens contribute to mammary tumor initiation and progression. According to the American Cancer Society, more than 70% of newly diagnosed cases of breast cancer are at their initial stages hormone-dependent [10], playing estrogens a crucial role in tumor genesis and progression. In this situation, estrogen receptor alpha is usually overexpressed. We refer to this class of tumors as hormone receptor-positive breast cancer. Since 1896, when Beatson reported the observation of regression of advanced breast cancer after bilateral ovariectomy in premenopausal women [11], there is considerable evidence pointing to estrogens as mammary carcinogens [12].

In 1978, Cohen et al. proposed that a diminished function of the pineal gland might promote an increase in the risk of breast cancer, as a consequence of a prolonged time of exposure to circulating estrogens. This hypothesis is based on several observations: (i) the incidence of breast cancer is lowest in countries in which pineal calcification shows a low incidence. (ii) Patients taking chlorpromazine, a drug that raises melatonin levels, have lower rates of breast cancer. (iii) *In vitro* data suggests that melatonin may have direct effects on breast cancer cells. (iv) Melatonin receptors are present on human ovarian cells, which suggest that melatonin may have a direct influence on the ovarian production of estrogen [13].

Plasma melatonin levels were determined in women with clinical stage I or II breast cancer. The amplitude of the night-time peak of nocturnal plasma melatonin was diminished in women with estrogen receptor-positive breast cancer in comparison with estrogen-negative disease patients or healthy matched control subjects, suggesting that low concentrations of melatonin during the night may increase the risk of hormone-dependent breast malignancy [14]. In 1987, based on Cohen's work, Richard Stevens suggested the hypothesis that women who are exposed to light-at-night (LAN) will have higher rates of breast cancer [15].

In 2001, Schernhammer et al. established a relation between rotating night-shift work and breast cancer risk in a cohort of premenopausal nurses, particularly in those women who reported more than 20 years of rotating night shifts [16]. Some other studies have evaluated the association between residential outdoor light during sleeping time with breast and prostate cancer among subjects that never worked at night, concluding that both prostate and invasive breast cancer were associated with high exposure to outdoor LAN [17, 18]. All these results support the hypothesis that, in healthy premenopausal women, LAN exposure can result in enhancement of mammary oncogenesis through disruption of the circadian oncostatic actions of melatonin.

The majority of *in vivo* studies performed to elucidate the role of melatonin in estrogen-responsive breast cancer have used as a model that chemically induced dimethylbenz(a)anthracene (DMBA) or N-methyl-N-nitrosourea mammary (NMU) carcinoma in young rats. Pinealectomized rats treated with DMBA and kept in short photoperiods (LD 10/14) showed a higher tumor incidence and a shorter latency period [19]. In contrast, animals with enhanced pineal function or receiving exogenous melatonin at pharmacological concentrations (dose: 500 mg/day, 2 weeks prior to DMBA during 20 weeks, injected at late afternoon) as the treatment had a decrease in the number of tumors. Additionally, an increase in tumor latency and a lower tumor incidence, generally accompanied by a smaller size and a higher frequency of tumor regression in previously induced tumors was obtained [20]. Moreover, the rodents treated with DMBA and receiving melatonin showed a reduced expression of estrogen receptor alpha at the tumor level. Therefore, the results from these animal models suggest that melatonin may counteract the action of estrogens in tumor cells *in vivo.*

**Figure 1:**
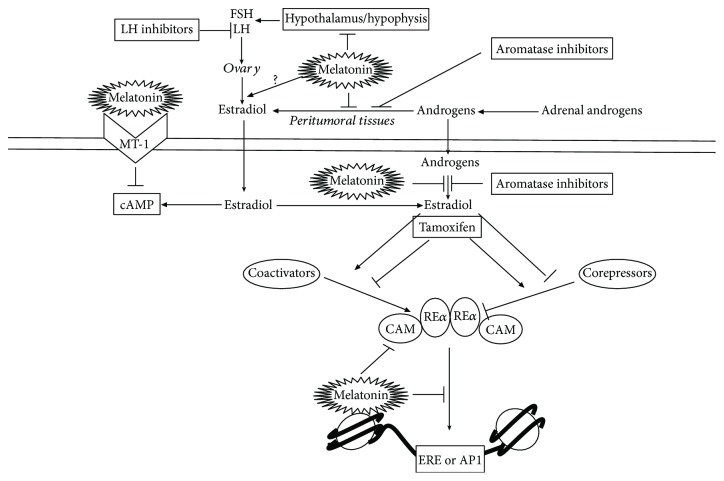
Melatonin antiestrogenic actions in estrogen-dependent human breast cancer cells: downregulation of the neuroendocrine/reproductive axis. Inhibition of the enzymes necessary for the conversion of adrenal androgens into estrogens in tumor surrounding tissues and in estrogen-dependent breast cancer cells. Inhibition of adenylate cyclase through binding to the MT-1 membrane receptor in epithelial malignant cells, counteracting the stimulatory effect of estradiol. Binding to the calmodulin-ER*α* complex, provoking destabilization of the estradiol-ER*α* complex, preventing its binding to DNA in both estrogen response element- (ERE-) and activator protein 1- (AP1-) containing promoters (whereas tamoxifen directly binds to the estrogen receptor, interfering with the binding of coactivators).

MCF-7, the first hormone-responsive breast cancer cell line has been widely used as an *in vitro* model of mammary tumors. The antiproliferative actions of the pineal hormone at the physiological nocturnal concentration (1 nM) on the breast cancer cell line MCF-7 have been studied for nearly thirty years [21]. There is abundant evidence suggesting that the inhibitory action of melatonin on mammary cancer estrogen-positive cell lines is based on its ability to regulate either the synthesis of estrogens or estrogen signaling pathways [22]. Concerning the antiestrogenic actions of melatonin, it is clear that the pineal hormone behaves as a selective estrogen receptor modulator (SERM). Whereas other antiestrogenic molecules used in clinical, such as tamoxifen, function as a selective antagonist for estrogen receptor alpha, in such way that in the 4-hydroxytamoxifen-hER alpha complex, helix 12 occludes the coactivator recognition groove [23] melatonin does not bind, at least directly, to the estrogen receptor. It has been described that a dose of melatonin equivalent to the physiological concentration found in plasma at night (1 nM) decreases the levels of ER*α* in *in vitro* experiments in MCF-7 cells [24]. Additionally, melatonin interferes with estradiol-triggered transcriptional activation of many estradiol-responsive genes through destabilization of the estradiol-ER complex, preventing its binding to DNA in both estrogen response element- (ERE-) and activator protein 1- (AP1-) containing promoters [25]. These actions of melatonin are likely mediated by calmodulin, since calmodulin binds to ER*α* and melatonin behaves as a calmodulin antagonist. The pineal hormone promotes structural changes in the calmodulin-ER*α* protein complex, thus impairing its binding to estrogen-responsive promoters [26, 27]. Due to these differential actions of melatonin and tamoxifen on the estrogen receptor, both molecules can cooperate; thus, it has been documented that physiological doses of melatonin (range from 2.32 pg/l to 23.2 ng/l) enhances the sensitivity of MCF-7 cells to tamoxifen [28]. Additionally, the SERM actions of melatonin can be explained since breast cancer cells express the melatonin receptor type 1 (MT-1) located in the membrane (Figure 1); the binding of melatonin to the MT-1 receptor results in inhibition of adenylate cyclase, therefore decreasing cAMP intracellular levels [29]. Conversely, estradiol activates adenylate cyclase, which results in higher cAMP cytoplasmic levels in a classical short-time second-messenger mechanism independent of gene transcription. Higher cAMP levels cooperate with long-time genomic effects of estradiol, thus enhancing ER-dependent transcriptional activation [30]. It is clear then that estrogens and the pineal hormone signaling pathways converge and have opposite effects over cAMP intracellular concentrations (Figure 1).

The oncostatic action of melatonin on hormone-dependent breast cancer cell lines is also based on its ability to limit the production of estrogens. Melatonin downregulates both the transcription and activity of many of the enzymes involved in the synthesis of estrogens, therefore behaving as a selective estrogen enzyme modulator (SEEM). Melatonin regulates both the transcriptional levels of steroid sulfatase (STS) and 17*β*-hydroxysteroid dehydrogenase type 1 (17*β*-HSD1), enzymes necessary to convert inactive estrogen precursors into physiologically active estradiol; estrogen sulfotransferase (EST), the enzyme that inactivates estrogens through their conversion to inactive estrogen sulfates; and aromatase, the enzyme responsible for the aromatization of androgens into estrogens (Figure 1). The regulation of melatonin takes account not only in tumor cells but also in endothelial and fibroblast cells located at the tumor cells adjacent tissues [31–36]. Interestingly, whereas the inhibitory effect of melatonin in MCF-7 cells is achieved at 1 nM, in *in vitro* experiments performed using fibroblasts or endothelial cells, a pharmacological dose of 1 mM is required. It has been reported that cytokines synthesized and released by tumor cells influence the surrounding fibroblasts, regulating their differentiation and stimulating both the aromatase expression and activity in them; melatonin at pharmacological concentrations (1 mM) counteracts this stimulatory effect, by inhibiting cytokines release from malignant cells (such as IL-6) and through the inhibition of the activity of aromatase [37, 38]. The vascular endothelial growth factor (VEGF) released by estrogen-responsive breast cancer cells binds to its receptor expressed in the membranes of adjacent endothelial cells. As a result, endothelial cells undergo proliferation, migration, and reorganization in a typical process of angiogenesis. Melatonin (1 mM) can regulate the paracrine mechanisms responsible for the interplay between mammary tumor cells and surrounding fibroblasts and endothelial cells in experiments performed *in vivo*, by downregulating the levels of VEGF and by inhibiting the production of aromatase in those cell lines [39, 40].

The *in vitro* anticancer actions of nocturnal human blood melatonin levels (1 nM) on hormone-dependent breast cancer cell lines can partially be explained since melatonin counteracts mitosis stimulated by estradiol [41]. Several protooncogenes (such as c-Myc) growth factors (TGF*α*) and transcription factors (NF-k*β*) targeted by the estrogen signaling pathways are modulated by this concentration of melatonin in breast cancer cells [42]. Similar results (at pharmacological doses) have been obtained *in vivo*, in Balb/c nude athymic mice (melatonin dose: 40 mg/kg of body weight, 5 days/week during 21 days) [43]. Estradiol and estrogen-like endocrine disrupting chemicals promote cell-cycle progression by increasing the levels of cell-cycle-related proteins such as cyclin D1 and cathepsin D, whereas it decreases the levels of p21 [44–46]. Melatonin (1 nM) inhibits cell-cycle progression causing a time delay in the G_1_-S transition, probably through an increase in the levels of p53 and p21 [47, 48]. The accumulation of cells in G_1_ forces them to enter into the G_0_ phase, inducing the endocrine-responsive cancer cells to undergo a higher differentiation. Additionally, it has been described that estrogens downregulate proteins implicated in adhesion such as E-cadherin and beta-(1) integrin. Melatonin (1 nM) increased the expression of these two cell surface adhesion proteins, leading to inhibition of cancer invasion and metastasis via ROCK- (Rho-associated protein kinase-) regulated microfilament and microtubule organization [49]. In tumor invasion, downregulation of adhesion proteins frequently occurs, leading to the loss of cell-to-cell recognition and transformation into a more aggressive invasive phenotype [50].

Melatonin is a promising agent to be considered as an adjuvant to chemotherapy and radiotherapy or to be combined with other molecules with anticancer activity in breast cancer treatment protocols. In humans, it has been reported that breast cancer patients receiving melatonin (a therapeutic dose: 20 mg/day in the evening administered for at least two months) during chemotherapy showed less side effects than matched controls receiving only chemotherapy [51]. Back to *in vitro* experiments, when a physiological dose of melatonin (1 nM or 10 nM) was combined with valproic acid, the melatonin MT-1 receptor expression was upregulated and the antiproliferative effect of valproic acid was more effective [52]. Melatonin (at a pharmacological dose, 1 mM) and troglitazone (a peroxisome proliferator-activated receptor-gamma agonist) together induced apoptosis in the breast cancer cell line MDA-MB-231 [53]. *In vivo*, the combination of pharmacological doses of melatonin (500 mg/day, 2 weeks prior to DMBA during 20 weeks, injected at late afternoon) and resveratrol reduced tumor incidence and decreased the quantity of invasive and in situ carcinomas in a rat model of experimental estrogen-responsive mammary carcinogenesis [54]. In a similar model of NMU-induced mammary tumors in rats *in vivo*, melatonin (dose: 20 *μ*g/ml in water starting 7 days prior to DMBA and kept for 16 additional weeks) potentiated the antitumor effect of pravastatin, further decreasing tumor frequency and lengthening tumor latency of this statin [55]. Exposure to dim LAN induced a circadian disruption of nocturnal melatonin in nude rats bearing estrogen receptor alpha-positive MCF-7 human breast cancer xenografts and consequently, a complete loss of tumor sensitivity to doxorubicin, indicating that chemotherapy resistance might be due to a disruption of the circadian structure ultimately leading to low levels of melatonin at night [56]. Combined with doxorubicin, the pineal hormone enhanced cancer cells apoptosis *in vivo* in rats bearing breast tumors. The animals treated with both doxorubicin plus melatonin (10 mg/kg of body weight, daily injected during 15 days) had the highest one-month survival rate and the lighter tumor weights [57]. *In vitro*, in MCF-7 cells, doxorubicin and melatonin had a synergic effect on apoptosis and mitochondrial oxidative stress. The apoptosis level, procaspase-9, PARP, caspase-3, and caspase-9 activities were higher in the group receiving both compounds at the same time (melatonin dose: 0.3 nM) [58]. Nocturnal human blood melatonin levels (1 nM) enhanced the antiproliferative and apoptotic responses to low doses of docetaxel in MCF-7 cells, modulating the changes in gene expression induced by this taxane. Docetaxel downregulated the mRNA levels of p53, cyclin-dependent kinase inhibitor 1A (CDKN1A), and cadherin-13 and upregulated mucin1 (MUC1), GATA-binding protein 3 (GATA3), and c-MYC, whereas melatonin reverted this actions. Moreover, *in vitro,* melatonin enhanced the expression of BAD and BAX and the inhibition of BCL-2 induced by docetaxel [59].

Ionizing radiation is a highly effective way of destroying any cancer cells remaining in the breast after surgery. Radiotherapy can cause a variety of adverse events in normal tissues leading to syndromes such as acute radiation (ARS) and multiorgan dysfunction syndromes (MODS) [60]. Radiation induces cellular damage by a variety of direct and indirect mechanisms ultimately resulting in DNA damage and chromosomal aberrations. The finding of new molecules acting as radioprotectors of normal tissues and organs is of great interest, and melatonin is a good candidate to be a radioprotective agent due to its hydroxyl radical scavenging ability [61]. Importantly, a physiological dose of melatonin (1 nM) decreased the ability of tumor cells to repair radiation-induced alterations of the DNA structure *in vitro*, mainly double-strand breaks (DSBs) that direct the cancer cells to undergo apoptosis. It is a common fact that cancer cells have abnormal high levels of proteins necessary for repair mechanisms. Thus, when the double-strand repair proteins such as RAD51 were downregulated, cells became more sensitive to radiotherapy [62]. Conversely, high levels of DNA-PKcs, a key player in nonhomologous end joining, accelerated the radiotherapy induced DSB repair mechanism [63].

*In vitro* experiments performed with leukemia cells showed that melatonin at a pharmacological dose (1 mM) increased the rate of apoptosis induced by radiotherapy, which is an effect that seemed to be dependent of p53 [64]. In the estrogen-dependent MCF-7 breast cancer cell line, melatonin treatment at physiological and pharmacological doses (1 nM, 10 *μ*M, 1 mM) prior to radiation resulted in inhibition of cell proliferation, induction of cell cycle arrest, and downregulation of RAD51 and DNA-PKcs [65]. Additionally, the levels of p53 were much higher in pretreated (melatonin 1 nM) cells [66]. As mentioned above, melatonin is an antiestrogen agent able to sensitize breast cancer cells to radiotherapy, and similar results have been obtained with classical aromatase inhibitors administered to breast cancer patients treated with ionizing radiation [67].

## 3. Melatonin and Hormone-Dependent Prostate Cancer

Prostate cancer (PC) is one of the leading causes of death by cancer among males in the developed world. As prostate physiology is under the control of androgens (testosterone and dihydrotestosterone) and their metabolites, androgen deprivation therapy (by inhibition of hormone biosynthesis or androgen receptor deprivation) has been extensively used in PC. However, most patients will develop hormone-refractory cancer [68]. As well as in the case of the mammary gland, the role of melatonin in the prostate has been established a long time ago. *In vivo*, melatonin (at a pharmacological dose of 150 mg/100 g of body weight, administered for 4 weeks) induced a significant decrease in the ventral prostate weight in castrated and castrated-testosterone-treated rats [69]. Conversely, pinealectomy was effective in stimulating androstenedione and testosterone production indicating an inhibitory action of the pineal gland on testicular steroidogenesis in rats [70]. Shortly after these findings, the involvement of melatonin in prostate cancer was unrevealed. In humans (patients with nonmetastasizing carcinoma), the pineal hormone did not show significant circadian rhythms indicating that the modulation of melatonin plasma levels might be related to prostate cancer genesis and growth [71]. Because of the antigonadotropic effect of melatonin, the hypothesis that the pineal hormone could inhibit prostate cancer was tested *in vivo* in rat prostatic adenocarcinoma. The main conclusion was that melatonin (50 *μ*g/rat, daily injected one hour before darkness) suppressed the growth of prostate tumors [72]. Back to humans, when the circadian rhythms of melatonin and 6-sulfatoxymelatonin were analyzed in the serum of elder patients with primary prostate cancer, a reduced pineal activity was found [73]. Also in humans, a clinical combination of melatonin and the LHRH analogue triptorelin was tested in metastatic prostate cancer patients with promising results. The concomitant administration of melatonin (a therapeutic dose, orally administered at 20 mg/day, in the evening every day until progression, starting 7 days prior to triptorelin) may overcome the resistance to LHRH analogues and palliate the adverse side effects [74]. According to these results, a physiological dose of melatonin (1 nM) attenuated the growth of the human androgen-sensitive prostatic tumor cell line LNCaP *in vitro* [75]. The expression of the membrane Mel1a melatonin receptor was demonstrated in this cell line, and an accumulation of the cells in G_0_/G_1_ and a decrease in S phase were obtained by treatment with melatonin at nanomolar concentrations [76]. Interestingly, the direct oncostatic activity of melatonin (1 nM) was also demonstrated *in vitro* in human androgen-independent DU 145 prostate cancer cells. As occurred in breast cancer, the indoleamine caused cell-cycle withdrawal by accumulation of cells in G_0_/G_1_ and inhibition of cell proliferation [77]. Back to the androgen-responsive prostatic LNCaP cells, a melatonin-mediated nuclear exclusion of the androgen receptor (AR) was demonstrated, indicating that melatonin (used at concentrations ranging from 1 nM to 100 nM) might regulate AR activity [78]. At 100 nM, melatonin induced a rise in intracellular cGMP, leading to an increase in calcium levels and PKC activation [79]. *In vivo* experiments in rodents showed that epidermal growth factor (EGF) stimulated LNCaP tumor growth in nude mice and induced an increase in the levels of Cyclin D1, whereas melatonin (at a pharmacological dose of 4 mg/g of body weight, administered intraperitoneally 1 h before lighting was switched off) counteracted this effect [80]. An *in vitro* study evaluating the effect of melatonin in both androgen-dependent (LNCaP) and androgen-independent (PC3) cells demonstrated that treatment with the indoleamine (10 nM to 1 mM) dramatically reduced the number of both types of cells and, in addition, induced cellular differentiation. The effect of melatonin was not mediated by PKA although a transitory rise in cAMP levels was observed [81]. In androgen-dependent prostate cancer cells, it has been demonstrated that pharmacological doses of melatonin (ranging from 50 nM to 1 mM) increased p21 levels, decreased NF-*κ*B activation, and Bcl-2 and survivin were downregulated [82]. The inhibition of NF-*κ*B signaling via melatonin-dependent activation (melatonin dose: 10 nM) of PKA and PKC resulted in transcriptional upregulation of p27 (Kip1), a MT1-dependent antiproliferative signaling mechanism [83]. In LNCaP cells, the pineal hormone (at different concentrations from 0–3 mM) induced both early and late apoptosis, both dependent of activation of c-JUN kinase (JNK) and p38 kinase, strongly suggesting that these kinases directly participate in apoptosis triggered by melatonin [84]. In androgen-dependent but also in androgen-independent prostate cancer cell lines, melatonin (1 mM) seemed to exert an antiangiogenic effect since it reduced hypoxia-inducible factor (HIF-1) protein levels and the release of the vascular endothelial growth factor, which correlated with dephosphorylation of p70S6 kinase and its target, ribosomal protein RPS6 [85]. In prostate cancer cell lines (*in vitro*) and transgenic adenocarcinoma or mouse prostate (TRAMP) mice (*in vivo*), melatonin (dose: 10–20 mg/l in drinking water, for 18 weeks) inhibited tumorigenesis by decreasing the serum levels of IGF-1, IGFBP3, and proliferation markers such as PCNA and Ki-67. Sirt1, a NAD(+)-dependent histone deacetylase overexpressed in prostate cancer, was also inhibited by melatonin in correlation to a significant antiproliferative effect [86]. *In vitro*, in both androgen-sensitive LNCaP and insensitive PC-3 cell lines, the pineal hormone (1 mM) limited glycolysis, the tricarboxylic acid cycle, and the pentose phosphate pathways, indicating that melatonin slows down glucose metabolism in both prostate cancer cell lines [87]. Yet more molecular mechanisms have been recently characterized *in vitro* (prostate cancer cell lines) and *in vivo* (TRAMP mice models). KLK2, KLK3 (kallikreins), and IGF1R were downregulated, whereas IGFBP3 was upregulated by melatonin (pharmacological doses: 10–20 mg/l in drinking water, for 18 weeks), demonstrating the role of the IGF signaling pathways in the oncostatic effect of the pineal hormone [88]. Additionally, an increase in phosphorylation of Akt in melatonin-treated (dose: 18 i.p. injections at 1 mg/kg of body weight, treatment lasting for 41 days) nude mice in which LNCaP cells were xenografted has been demonstrated *in vivo* [89]. Recent evidence suggests that microRNAs (miRNAs) are good candidates to be considered as targets in cancer treatments. In androgen insensitive PC-3 lines subjected to hypoxia, melatonin (1 mM) upregulated miRNA3195 and miRNA374b. Overexpression of miRNA3195 and miRNA374b decreased the levels of the proangiogenic proteins VEGF, HIF-1, and HIF-2, thus explaining, at least in part, the antiangiogenic properties of melatonin in prostate cancer [90]. It is widely accepted that desynchronization of the clock circuitry after alterations in the circadian rhythms is implicated in cancer. Indeed, melatonin (100 *μ*M to 2 mM) increased the levels of Per2 and Clock, whereas reduced Bmal1 in prostate cancer cells [91]. Combined with chemotherapeutic agents (etoposide, doxorubicin, or docetaxel), melatonin (1 mM) enhanced the sensitivity of cancer cells to cytokine-induced apoptosis *in vitro* [92].

As in breast cancer, some attention has been drowned into the hypothesis that light-at-night (LAN) exposure can inhibit nocturnal melatonin, and consequently, prostate cancer risk would be elevated. The countries with higher levels of nocturnal light yielded a higher risk of prostate cancer [93]. Men who never worked at night in night-shift turns have a lower risk of prostate cancer in comparison with night-shift workers [94]. Men who reported sleep problems had lower morning levels of urinary 6-sulfatoxymelatonin in association with an increased prostate cancer risk [95]. Aggregate genetic variation in melatonin and circadian rhythms were also significantly associated with the risk of prostate cancer, but no significant association could be established for lung and ovarian cancer, supporting a potential role of melatonin pathways and circadian rhythms in prostate carcinogenesis [96].

## 4. Melatonin and Ovarian Cancer

Ovarian cancer (OC) is the main cause of death in the world when speaking about gynecological malignancies. Sexual hormones (estrogens, progesterone, and testosterone) seem to increase the risk of ovarian cancer. The use of oral contraceptives has a strong protective association with ovarian cancer risk. Parity correlates with lower risk, with a greater reduction for each additional pregnancy. However, menopause at late ages increases the risk of OC [97]. Another study in pregnant women indicates that higher concentrations of testosterone and 17-hydroxy-progesterone increase the risk of borderline serous tumors; if androstenedione is elevated, the risk of mucinous tumors is increased; in conclusion, higher than average concentrations of estradiol increase the risk of endometrioid tumors [98].

At first, melatonin, in *in vitro* experiments, (range from 20 to 200 *μ*M) was found to be the only neuroendocrine hormone that stimulated cell proliferation of the KF cell line, derived from human serous cystadenocarcinoma [99]. Despite this negative result, many others investigated the effects of melatonin in OC. As melatonin is synthesized by granulosa cells of preovulatory follicles, it has been attributed as a role influencing sex steroid hormones production essential for ovulation [100]. After this hypothesis, many works related, at least indirectly, a melatonin-deficient production with lower levels of progesterone and higher levels of estradiol. After menopause, melatonin secretion dramatically decreases in correlation with most frequent anovulatory cycles and increased risk of endometrial carcinoma [101].

As aforementioned, prostate and breast cancer have been associated with night-shift work. For these sexual hormone-related cancers, the higher risk in people exposed to LAN might be explained in terms of cessation of melatonin production. However, there was no association between rotating night-shift work and risk of ovarian cancer [102]. Additionally, when urinary 6-sulfatoxymelatonin (aMT6) was measured in ovarian cancer and healthy women samples, the results showed that aMT6 was not significantly associated with risk of OC [103]. Conversely, another recent study, in which melatonin was measured in serum from women with ovarian cancer and healthy controls, a significant reduction in serum melatonin levels was found in ovarian cancer patients [104].

Despite these apparently discrepant results, melatonin (at a tentative therapeutic dose: 40 mg/day orally taken, weeks until progression) was tested in humans (advanced ovarian cancer patients) in combination with IL-2. Although the number of cases was too low to establish solid conclusions, the combination of low-dose IL-2 plus this therapeutic dose of melatonin showed some promising results [105]. Since melatonin had been previously reported to be effective in reducing proliferation in many cell lines derived from different types of cancer, the pineal hormone was tested *in vitro* in the estrogen-dependent BG-1 ovarian adenocarcinoma cell line. Melatonin at physiological concentrations (1 to 100 nM) caused a reduction in cell number, indicating an oncostatic action of the indoleamine [106]. The melatonin MT-1 membrane receptor is likely to be implicated in the pineal hormone effect, since it is expressed in both normal ovarian epithelial and ovarian cancer cell lines. As previously described in breast cancer cells, binding of melatonin to its receptors in OC cell lines inhibits adenylate cyclase, therefore reducing the levels of cAMP [107, 108].

In rodents, in an *in vivo* model of chemically induced ovarian carcinomas in ethanol-preferring rats, melatonin treatment (dose: 200 *μ*g/100 g of body weight, administered from 60 days) reduced ovarian masses and the incidence of adenocarcinomas in ethanol deprived rats [109]. When the molecular mechanisms underlying this effect were explored, it was revealed that melatonin administration suppressed the increase in the levels of Her-2, p38, phospho-AKT, and mTOR that usually take place in OC [110]. Other proteins whose levels are usually increased in OC were diminished by melatonin: Toll-like receptor 4 (TLR4), MyD88, IkK*α*, NF-*κ*B, TRIF, and IRF-3, all involved in MyD88- and TRIF-dependent signaling pathways induced by TLR4 [111]. In the same ethanol-preferring rat model, melatonin promoted apoptosis through the upregulation of p53, BAX, and caspase-3 and downregulation of Bcl-2 and survivin [112]. A proteomic analysis performed in animals receiving long-term melatonin showed that the pineal hormone induced downregulation of several proteins involved in metabolic processes, generation of metabolites, hypoxia signaling, endoplasmic reticulum stress-associated proteins, and cancer-related proteoglycans. A few proteins were upregulated; fatty acid-binding protein (FABP), mitochondrial heat shock protein 10 (hsp10), or the product of the gene ATP5F1B, which is ATP synthase subunit *Β* [113]. In the same *in vivo* model, melatonin also inhibited angiogenesis, which is a process that occurs in many types of cancer, including OC. In ethanol-preferring rats with serous papillary OC, VEGF, the key signal to induce the formation of new vessels, is downregulated by the pineal hormone [114]. *In vitro*, in the OC cell lines OVCAR-429 and PA-1, melatonin treatment (0–800 *μ*M) resulted in an accumulation of cells in the G_1_ phase of the cell cycle in parallel with a downregulation of CDK2 and CDK4, another finding that contributes to explain the antitumor activity of melatonin in ovarian cancer [115]. Invasion and metastasis are two processes that allow tumor cells to spread throughout the body. In cancer stem cells obtained from ER*α* (−) SK-OV-3 ovarian cancer cells, melatonin inhibited proliferation (as seen by an important decrease in the proliferation marker Ki-67). Additionally, ZEB1, ZEB2, vimentin, and snail, genes related to epithelial-to-mesenchymal transition, were decreased after the indoleamine treatment. Migration of cancer stem cells was also inhibited by melatonin (3.4 mM), indicating that the pineal hormone might be an important adjuvant to prevent invasion and metastasis [116].

Recently, many researchers have focused their attention on the potential benefits that melatonin might have modulating the actions of chemotherapeutic agents [6]. Most of the studies testing the ability of melatonin to enhance the antiproliferative effects of chemo in OC have been performed combining melatonin with cisplatin. In a pioneer chemosensitivity assay performed *in vitro*, melatonin (0.1 nM to 10 nM) proved to be more efficient than cisplatin inhibiting the cell growth of primary cells from ovarian tumors [117]. In OC HTOA cells (sensitive to cisplatin) and OVCAR-3 (resistant to cisplatin), melatonin (1 nM and 1 *μ*M) did not have an antiproliferative effect when applied alone but enhanced the sensitivity of cisplatin. Additionally, telomerase activity was lower in the OVCAR-3 cell line treated with this indoleamine [118].

The apparent inefficacy of melatonin (range from 0 to 2 mM) to impair by itself the growth of ovarian cancer was confirmed in SK-OV-3 cells. However, when combined with cisplatin, melatonin synergistically cooperate with the chemo drug to diminish the viability in this cell line, effect accompanied by an increase in the cleavage of PARP and caspase-3, an inhibition of ERK phosphorylation along with dephosphorylation of p90RSK and HSP27. Importantly, melatonin showed a protective effect against cisplatin toxicity in OSEN noncarcinogenic ovarian epithelial cells [119].

The pineal hormone protective effect was also observed *in vivo*; in mice, pharmacological doses of melatonin (ranging from 15–30 mg/Kg of body weight, i.p. injected 3 days) prevented from cisplatin-induced loss of the follicle reserve, likely through an inhibition of the activation of PTEN/AKT/FOXO3a signaling pathway [120]. Melatonin, at the same doses, in combination with ghrelin in cisplatin-treated ovaries increased the affinity of FOXO3a to p27kip promoter, restoring its expression, which is critical to maintain the dormant status of primordial follicles using an ex vivo ovary culture system [121]. *In vivo*, this protective action was abolished in mice (pharmacological doses of melatonin ranging from 5–20 mg/kg of body weight, i.p. injected 3 days prior to cisplatin) when the intracellular signaling triggered by the MT-1 receptor was blocked, indicating that the cytoprotective effect of the indoleamine is mediated by the MT-1 membrane receptor [122]. However, melatonin (0.1 mM to 2 mM) enhanced the cytotoxic effect of cisplatin *in vitro* in three independent OC cancer cell lines in an MT-1 independent manner [123]. Additionally, melatonin (range from 0 to 10 mM) further stimulated apoptosis induced by laser treatment in OC tumor cells [124].

## 5. Melatonin Influences Breast, Prostate, and Ovary Cancer at Different Levels

### 5.1. Light-at-Night and the Risk of Breast, Ovarian, and Prostate Cancer

Light-at-night (LAN) exposure impairs the nocturnal peak of melatonin, and consequently, the pineal gland hormone cannot reduce the production of estrogens and androgens. These sexual hormones, together with progesterone, control the normal physiology of the breast, the prostate gland, and the ovary; however, augmented levels of these hormones can increase the incidence of breast, prostate, and ovarian cancer. Therefore, it was proposed that an increased exposure to nocturnal light might result in a higher risk of endocrine-related cancers. As aforementioned, there is a clear association between rotating night-shift work and breast cancer risk, supporting the hypothesis that LAN exposure, in healthy premenopausal women, can enhance mammary oncogenesis through disruption of the circadian rhythms [17–19]. In men, there is a clear association of LAN with a higher risk of prostate cancer [93–96]. However, in ovarian cancer, such an association has not been established [102].

### 5.2. Melatonin Levels in Patients with Hormone-Dependent Cancers

The amplitude of the nocturnal peak of melatonin was decreased in women with estrogen receptor-positive breast cancer in comparison with healthy women [14]. Melatonin lost the circadian rhythmic production in men with prostate cancer as compared to healthy men [73], and some discrepant results have been obtained in the case of ovarian cancer. Whereas some studies reported no clear association between urinary melatonin and ovarian cancer risk [103], others found lower serum levels of melatonin in women with ovarian cancer compared to healthy matches [104].

### 5.3. Melatonin Receptors in Hormone-Dependent Cancers

Concerning the expression of the melatonin membrane receptors, in the breast, prostate, and ovarian cancer cells, the expression of the MT-1 receptor has been reported [29, 76, 83, 107, 108, 125, 126], indicating a potential prognostic and therapeutic significance of MT-1 in hormone-dependent cancers.

In breast cancer cells, melatonin and estradiol signaling pathways converge, since they have opposite effects over cAMP intracellular concentrations. The effect of the pineal hormone is dependent on its binding to the MT-1 melatonin receptor located in the membrane [29, 30]. A similar inhibitory effect of melatonin (dose range from 1–100 nM) on adenylate cyclase, therefore reducing the levels of cAMP, has been described in both ovarian cancer cells [107, 108] and ovarian granulosa cells (melatonin dose range from 0.1 *μ*M to 10 *μ*M) [127]. However, the regulation of AR activity in prostate cancer cells by melatonin is likely to be dependent of a rise in intracellular cGMP, leading to an increase in calcium and activation of PKC [78, 79].

### 5.4. Melatonin Regulates the Synthesis of Estrogens and Androgens in Normal and Cancer Tissues

The effect of melatonin on steroid production (estradiol is the natural growth factor of breast and ovarian cancers) by normal granulosa cells depends on the model chosen for the study: in ovine granulosa cells, the pineal hormone (tested at several concentrations from 0.86 to 86 nM) had no significant effect on the production of estradiol [128]. In porcine ovaries (doses range: from 1 pg to 100 ng/ml of the medium during 9 days) and human granulosa cultured cells, melatonin (from 10 nM to 1 mM) stimulated estradiol secretion [129, 130]. In contrast, in preovulatory follicles in the cyclic hamster, a clear inhibition of progesterone and estradiol production by melatonin (0.1–10 ng/l) was observed, likely through a reduction in the levels of cAMP [131]. Increased levels of estradiol have been found in women who worked 15 or more years of night shifts, a change that might be associated with an increased cancer risk [132]. Contrary to the discrepancies obtained in normal granulosa cells, it has been extensively demonstrated that the pineal hormone is an inhibitor of sexual hormones synthesis in breast cancer cell models. Melatonin downregulates some of the enzymes necessary for the synthesis of estrogens, such as aromatase (cytochrome P450), steroid sulfatase (STS), and 17b-hydroxysteroid dehydrogenase type 1 (17*β*-HSD1), whereas it stimulates estrogen sulfotransferase (EST), the enzyme that inactivates estrogens. Therefore, melatonin can be labelled as a selective estrogen enzyme modulator. Interestingly, the effect of melatonin takes account in both epithelial malignant breast tumor cells and adjacent peritumoral endothelial cells and fibroblasts; strikingly, melatonin is effective at nanomolar concentrations in breast cancer cells but only at millimolar concentrations in peritumoral cell models [31–36]. Steroid sulfatase (STS), estrogen sulfotransferase (EST), and 17b-hydroxysteroid dehydrogenases 2 (17BHSD2) and 5 (17BHSD5) have been detected in ovarian cancer cell lines, with a higher formation of estradiol in comparison to normal ovarian epithelium cells [133]. In ovarian cancer samples from patients, higher levels of estrogen inactivating enzymes are associated with ER*α* abundance and with a better overall survival rate [134]. To date, the potential inhibitory role of melatonin in these enzymes in ovarian cancer cells has not been tested.

Testosterone is the natural growth factor of prostate cancer. The inhibitory action of melatonin (10 pM to 1 *μ*M) on both testosterone-induced prostate growth and testosterone production by Leydig cells has been known for decades [135, 136]. This reduction of testosterone production by melatonin (10 pM to 1 *μ*M) likely takes account through the binding of the pineal hormone to the MT-1 receptor expressed in Leydig cells, resulting in decreased levels of cAMP [137]. In hamsters, melatonin (10 pM to 1 *μ*M) suppresses testicular steroidogenesis through inhibition of several enzymes, such as steroidogenic acute regulatory protein (StAR), cholesterol side-chain cleavage enzyme (cytochrome P450SCC), and 3*β*-hydroxysteroid dehydrogenase (3*β*-HSD) [137, 138]. In Leydig cell lines from mice, the reduction of testosterone production by melatonin (range from 1 pM to 1 *μ*M) is related to a decrease in cAMP levels and downregulation of GATA-4 and SF-1 transcription factors [139]. Back in human, higher levels of androgens and a delayed peak of androgen production have been described in night-shift workers [140]. Although androgens contribute to prostate hyperplasia and cancer, and androgen deprivation therapy has been used for decades, most patients will develop refractory cancer, probably by the coexistence of different populations of cells, some dependent and some not dependent of androgens [141]. Recent findings suggest that 3*β*-hydroxysteroid dehydrogenase is induced upon androgen stimulation via androgen receptor in prostate cancer cells, establishing an androgen production positive feedback loop [142].

### 5.5. Estrogen and Androgen Receptors Are Regulated by Melatonin

Additionally, to inhibition of androgen and estrogen production, melatonin can also control the intracellular levels and activity of the estrogen and androgen receptors. Therefore, the inhibitory action of melatonin on hormone-dependent cancer cells is primarily based on its ability to regulate estrogen and androgen signaling pathways; again, most of the research has been conducted in breast cancer models. The pineal hormone decreases the levels of ER*α* in MCF-7 cells [24] and destabilizes the estradiol-ER complex preventing its binding to estradiol-responsive promoters [25], likely through interaction with calmodulin bound to ER*α* [26, 27]. In prostate cancer cells, a melatonin-mediated nuclear exclusion of the androgen receptor has been described, indicating that melatonin might regulate the androgen receptor activity [78]. In ovarian cancer, the effect of the pineal hormone on the ER*α* levels remains unexplored, although it is known that melatonin (100 *μ*g/100 g of body weight/day, 150 days) decreases estradiol, increases progesterone, and downregulates androgen receptor, ER*α*, and ER*β* levels in oviducts and uteri of rats [143].

### 5.6. Melatonin Exerts Antiproliferative Effects and Induces Apoptosis in Breast, Ovarian, and Prostate Cancers

Melatonin at physiological concentrations (1 nM) exerts antiproliferative actions in breast, prostate, and ovarian cancer cells. A common fact after melatonin treatment is the accumulation of cells in the G_1_ phase of the cell cycle. In estrogen-responsive breast cancer cells, this effect is probably reached through an increase in the levels of p53 and p21 in parallel with a downregulation of cyclin D1; p53 participates in growth suppression and apoptosis; the activation of p21 by p53 leads to a failure in phosphorylation of the retinoblastoma factor and cell cycle arrest [144]. Therefore, cells are forced to enter into the G_0_ phase, undergoing a higher stage of differentiation [47, 48]. Interestingly, the accumulation of cells in G_0_/G_1_ after treatment with melatonin at nanomolar concentrations has been described in both estrogen-responsive and estrogen-independent breast cancer cells [145, 146] and also in androgen-sensitive and androgen-independent cancer cells [76, 77]. The stimulated levels of p53 in response to melatonin correlate with enhanced release of TGF*β*-1 in breast cancer [24] and prostate benign cells (melatonin dose: 10–500 *μ*M) [147]. In ovarian cancer, the effect of melatonin on the TGF*β*-1 pathway has not yet been addressed. Nevertheless, in ovary cancer, it is known that the increase in the percentage of cells in G_1_ in response to melatonin (range from 0 to 800 *μ*M) is associated with a downregulation of cyclin-dependent kinases 2 and 4 (CDK2 and CDK4) [115].

When cells accumulate in the G_0_ phase, they can differentiate or else they can undergo apoptosis. Melatonin (1 nM) promotes apoptosis in hormone-sensitive breast cancer cells [148], prostate cancer cells (melatonin dose: 0–3 mM) [84], and in an *in vivo* (rat) model of ovary cancer (melatonin dose: 200 *μ*g/100 g of body weight, administered from 60 days) [112]. Concerning apoptosis mediators, a significant increase in caspase-3 activity has been described in a breast cancer model developed in rats (melatonin administered from 2 weeks prior to DMBA for 3 months, at 250 mg/100 g of body weight) [149]. In MCF-7 cells, melatonin (1 nM) induced activation of caspases-9 and -7 and cleavage of PARP in parallel to a reduction of the Bcl-2/Bax ratio [148]. These results suggest that melatonin can trigger two apoptotic processes in MCF-7 cells: one TGFbeta1 and caspase-independent response and another process dependent of TGFbeta1 in which caspase-7 is the effector [148]. Melatonin also has proapoptotic effects in ER-negative breast cancer cells through inhibition of Cox-2, Akt, PI3K, p300, and NF-*κ*B signaling pathways, although this effect takes account at pharmacological doses (1 mM) [150]. The pineal gland hormone (0.3 nM) also enhanced apoptosis combined with chemotherapeutic agents such as doxorubicin in rats bearing mammary tumors. PARP, procaspase 9 and caspase-3, and caspase-9 activities were higher in the animals simultaneously treated with both compounds [58]. In the MCF-7 cells, melatonin (1 nM) enhanced the expression of BAX and BAD and reduced the levels of BCL-2 induced by docetaxel. In a model of ovarian carcinoma in rats, melatonin (dose: 200 *μ*g/100 g of body weight, administered from 60 days) inhibited the phosphorylation of AKT and mTOR [110], diminished the levels of TLR4, MyD88, IkK*α*, NF-*κ*B, TRIF, and IRF-3 [111], and promoted apoptosis through the upregulation of p53, BAX, and caspase-3 and downregulation of Bcl-2 and survivin [112]. In prostate cancer cells, the apoptotic effect of melatonin (0–3 mM) has also been observed, and the molecular mechanism does not differ much from those described in breast and ovary cancers: increased expression of p53, p27, and p21, fragmentation of PARP and activation of caspases-3, -8, and -9 [84, 151]. Bax expression was markedly activated and Bcl-2 inhibited; the apoptotic effect of melatonin involved activation of JNK and p38 [151].

### 5.7. Melatonin Inhibits Angiogenesis in Breast, Prostate, and Ovarian Cancers

The antiangiogenic activity of melatonin was described for the first time in metastatic patients. When melatonin was given orally at 20 mg/day during two months, a significant decline in VEGF secretion was observed [152]. An apoptotic and antiproliferative effect of melatonin (tested from 0.1 nM to 1 mM) has been described in human umbilical vein endothelial cells in association with upregulation of p53 and Bax and downregulation of Bcl-2 [153]. VEGF is released by estrogen-responsive cancer cells and binds to its receptor located in the membranes of adjacent endothelial cells. The pineal hormone, administered at pharmacological concentrations (1 mM), downregulated the levels of VEGF and the production of aromatase [39, 40]. In cocultures of breast cancer (MCF-7) and endothelial cells (HUVECs), melatonin (1 mM) exerted its antiangiogenic action by downregulating the expression of angiopoietins (ANG-1 and ANG-2) and VEGF levels and upregulating the expression of Tie2, which is the angiopoietin receptor [154]. Similar results have been obtained in xenograft models of hormone-dependent breast cancer (melatonin administered for 21 days at 40 mg/kg of body weight, 5 days a week) [155] and in triple-negative breast cancer cell lines (melatonin dose: 1 *μ*M to 1 mM) [156]. In androgen-dependent breast cancer, melatonin (1 mM) reduced the levels of HIF-1 and inhibited the release of VEGF [85]. Lower angiogenesis was found in melatonin-treated (18 i.p. injections of 1 mg/kg in 41 days) mice xenografted with LNCaP cells [89]. Melatonin (1 mM) upregulated miRNA3195 and miRNA374b in prostate cancer cells in parallel with a reduction of VEGF, HIF-1, and HIF-2 levels [90]. Melatonin also inhibited angiogenesis in ethanol-preferring rats with ovarian carcinoma. As in breast and prostate cancer, the proangiogenic factor VEGF was downregulated by the pineal hormone [114].

### 5.8. Melatonin Protection against Invasion and Metastasis

A pilot phase II study including patients with metastatic breast cancer concluded that the administration of melatonin (20 mg/day in the evening) might induce tumor regression [157]. Melatonin, at physiological concentrations (1 nM), reduced the invasiveness of hormone-dependent MCF-7 cells and increased the levels of E-cadherin and beta1 integrin [158]. Melatonin (1 nM) stimulated the Rho-associated protein kinase (ROCK-1) in MCF-7 cells [49] and in an *in vivo* model (female mice) of breast cancer (melatonin administered at 100 mg/kg of body weight, 5 days a week, up to 6 weeks) metastasis in the lung [159]. The pineal hormone activated glycogen synthase kinase 3*β* (GSK3*β*) by inhibiting Akt phosphorylation, inducing *β*-catenin degradation, and thus inhibiting epithelial-to-mesenchymal transition [160]. After treatment with 1 mM melatonin, MCF-7 cells reduced cell migration and invasion; E-cadherin expression was increased, whereas OCT4, N-cadherin, and vimentin were reduced [161]. In MCF-7 cells overexpressing Her2, melatonin (range from 0.1 pM to 1 *μ*M) repressed the epithelial-to-mesenchymal transition by suppressing RSK2 expression [162]. In ovarian cancer cells, ZEB1, ZEB2, vimentin, and snail, genes related to epithelial-to-mesenchymal transition, were downregulated after melatonin (3.4 mM) treatment [116].

### 5.9. Synergistic Actions of Melatonin in Breast, Prostate, and Ovarian Cancers

The protective role of melatonin against chemotherapy side effects is well documented; thus, in clinical trials, melatonin (20 mg/day orally every day at evening at least two months) enhanced the efficacy and reduced the toxicity of chemotherapy [163, 164]. The protective effect of melatonin (10 *μ*M) was also observed in hematopoietic stem cells treated with doxorubicin [165]. Patients receiving melatonin (daily 21 mg at bedtime) showed a reduction of taxane-induced neuropathy [166]. In rats, the pineal hormone (tested at 5/10/50 mg/kg of body weight, daily, starting 3 days prior to paclitaxel, during 5 days) protected against paclitaxel-induced neuropathic pain [167].

In breast cancer, melatonin has been tested in combination with many substances, and, in most cases, potential positive effects have been documented. In MCF-7 cells, melatonin 1 nM promoted apoptosis and/or inhibited cell proliferation in combination with all-trans retinoic acid [168], valproic acid (primarily used to treat epilepsy) [52], troglitazone (a peroxisome proliferator-activated receptor-gamma agonist) [53], somatostatin [169], or arsenic trioxide [170]. In animal models, treatment with melatonin (200 *μ*g/rat per day, 300 days) enhanced the chemoprophylactic effect of tamoxifen [171] and resveratrol (melatonin dose: 500 mg/day, 2 weeks prior to DMBA, during 20 weeks, injected at late afternoon) [54]. The pineal hormone administered by subcutaneous injection (500 *μ*g daily, 1 h before darkness, 20 weeks) potentiated the tumor prevention by 9-cis-retinoic [172]. At 20 mg/ml added in the water (7 days prior to the carcinogen, administered during 15 weeks), melatonin potentiated the antitumor effect of statins [55, 173].

Light-at-night (LAN) and subsequent melatonin disruption have been related to the development of resistance to tamoxifen [174] and doxorubicin [56]. The pineal hormone has also been tested in combination with chemotherapeutic agents. Thus, melatonin (0.3 mM) and doxorubicin had synergic effects on apoptosis in MCF-7 cells [58] and, at 1 nM, enhanced the antiproliferative and apoptotic effects induced by docetaxel [59].

In ovarian cancer, a pioneer study including a modest number of patients addressed the effect of melatonin (dose: 40 mg/day orally taken, weeks until progression) with IL-2, with some promising results [105]. However, to date, melatonin has not been used as an adjuvant at a clinical level [175]. In studies performed in ovarian cancer cell lines, melatonin (1 nM) and cisplatin synergistically cooperated, diminishing proliferation [118] and increasing apoptosis (cleavage of PARP and activation of caspase-3). These effects were independent of membrane MT-1 receptors [123]. Importantly, melatonin showed a protective effect against cisplatin toxicity *in vitro* (noncarcinogenic ovarian epithelial cells) [119] and *in vivo* (mice models, melatonin doses: 15–30 mg/Kg of body weight, i.p. injected 3 days) [120, 121]. This protective action is likely mediated by the MT-1 membrane receptor [122].

In prostate cancer, it has been described that a combination of melatonin (orally at 20 mg/day, in the evening every day until progression, starting 7 days prior to triptorelin) and the LHRH analogue triptorelin may overcome the resistance to LHRH analogues and palliate the adverse side effects [74]. Melatonin (1 mM) enhanced the apoptotic effects of etoposide, doxorubicin, or docetaxel in prostate cancer cells [92].

## 6. Conclusions and Remarks

Melatonin is a naturally produced hormone with high expectations to be included as an adjuvant in cancer treatments, although the intracellular mechanisms triggered by this indoleamine are not yet completely characterized. In breast, ovary, and prostate cancer, an inhibitory action of the pineal gland on sexual hormones steroidogenesis has been demonstrated. Additionally, melatonin regulates the levels and modulates the transcriptional activity of the estrogen (ER) and androgen (AR) receptors. Concerning estrogens, there is abundant evidence pointing to melatonin as a molecule able to regulate estrogens synthesis (acting as a SEEM) and estrogen receptor activity (acting as a SERM). Melatonin regulates enzymes involved in the synthesis of androgens as well as the androgen receptor activity. Exposure to light-at-night (which abolishes the nocturnal peak of melatonin) is associated with an increase in the risk of breast and prostate cancer, although the association with the risk of ovary cancer is not totally clear.

Some of the cellular processes regulated by the pineal hormone are gathered in Table 1. The molecular target genes which expression, translation, or posttranslational modifications are enhanced by melatonin are shown in Table 2, and those which expression, translation, or posttranslational modifications are downregulated by melatonin are shown in Table 3. The results have been compiled from research performed in various cancer-derived cell lines, animal models, and samples obtained from cancer patients. With practically no reports claiming the contrary, melatonin exerts antiproliferative actions in prostate, breast, and ovary tumors, by itself or enhancing the sensitivity to chemotherapeutic drugs.

Melatonin is a proapoptotic molecule, and some of the molecular targets involved in apoptosis found in the different cancer models eventually resulted to be the same in the three kinds of cancer. Breast, ovary, and prostate cancer cells undergo a delay in cell-cycle progression after melatonin treatment. The pineal hormone impairs the epithelial-to-mesenchymal transition, inhibits cell migration, invasion, and metastasis (either alone or in combination with chemotherapy). The pineal hormone also inhibits angiogenesis.

In many different types of cancer, melatonin has been combined with many antitumor agents, among them were tamoxifen, valproic acid, pravastatin, doxorubicin, epirubicin, docetaxel, etoposide, cisplatin, methotrexate, irinotecan, ursolic acid, 5′-fluorouracil, celecoxib, capecitabine, gemcitabine, cyclophosphamide, sorafenib, gefitinib, aracytin, puromycin, clofarabine, everolimus, barasertib, or temozolomide [6]. Additionally, melatonin has been tested in cells lines, animals, and even patients receiving radiotherapy.

The results obtained to date are really promising, since melatonin synergizes the chemotherapy effects, allows to use lower doses (which might result in better tolerance), and protects from the undesirable side effects of radiotherapy and most of the chemotherapeutic agents aforementioned. In summary, after additional molecular studies and newly designed clinical trials combining melatonin and either chemo- or radiotherapy have been conducted, it might be reasonable to consider the pineal hormone as a potential agent to be included in cancer treatments.

## Figures and Tables

**Table 1:** Association of light-at-night (LAN) and melatonin levels with the different types of cancer; melatonin actions on sexual hormones synthesis, proliferation, apoptosis, cell cycle, invasion, and synergistic effects with other molecules in different cancers; yes^1^: only described in one report; yes^2^: at least one contradictory study.

	LAN and cancer risk	Melatonin levels and cancer risk	Synthesis of sexual hormones	Antiproliferation	Apoptosis	Cell cycle	Antiangiogenesis	Invasion	Synergistic actions
Breast	Association	Inverse association	Inhibition	Yes	Yes	Delay G_0_-G_1_	Yes	Yes	Yes
Prostate	Association	Inverse association	Inhibition	Yes	Yes	Delay G_0_-G_1_	Yes	Not described	Yes^1^
Ovary	No association	Not described	Not tested	Yes^2^	Yes	Accumulation in G_1_	Not described	Yes	Yes

**Table 2:** Melatonin upregulated and/or activated molecular targets in breast, prostate, and ovary cancer.

	Synthesis of sexual hormones	Antiproliferation	Apoptosis	Cell cycle	Antiangiogenesis	Invasion-metastasis
Breast cancer	EST	p53, p21	p53, p21, caspase3, caspase7, caspase9, PARP, Bax, Bad, TGFb-1	p53, p21	Tie-2	E-cadherin, beta1-integrin, ROCK, cadherin13, GSK3*β*,
Prostate cancer		p53, p21, Kip1, IGFBP3, Per2, Clock, pAKT	p53, p21, Kip1, JNK, p38, PKC, caspase3, caspase8, caspase9, TGFb-1, PARP, Bax	p53,	miRNA3195, miRNA374b, HIF-1	
Ovary cancer		p53,	p53, caspase3, Bax, PARP	p53,		FABP, ATP5F1B, HSP10

**Table 3:** Melatonin downregulated and/or inhibited molecular targets in breast, prostate, and ovary cancer. ^1^: indirect effect on estradiol synthesis in adipocytes.

	Synthesis of sex steroids in gonads or tumoral tissues	Antiproliferation	Apoptosis	Cell cycle	Antiangiogenesis	Invasion-metastasis
Breast cancer	STS, 17b-HSD1, aromatase, (IL-6, IL-11, TNF-*α*)^1^	MUC1, GATA3, c-myc, TGF*α*, ER*α*, cyclin D1, COX-2, AKT, PI3K, p300, NF-*κ*B	NF-*κ*B, Bcl-2, RAD51, DNA-PKcs, COX-2, AKT, PI3K, NF-*κ*B p300,	Cyclin D1	VEGF, ANG-1, ANG-2	p38, MMP-2, MMP-9, pAKT, OCT4, Ncadherin, vimentin, RSK, *β*-catenin
Prostate cancer	StAR, cytochrome P450SCC, 3*β*-HSD, GATA-4, SF-1	AR, Sirt1, KLK2, KLK3, IGF1R, Ki-67, PCNA, BmalI, NF-Kb	NF-kB, Bcl-2, survivin	Cyclin D1	VEGF, HIF-1, HIF2, p70S6K, RPS6	KLK2, KLK3
Ovary cancer	Estradiol secretion stimulated/inhibited depending on the model	p38, pAKT, Her-2, mTOR, MyD88, interferon *β*, pERK, p90RSK, pHSP27, IRF3, Ki-67	NF-kB, TLR4, IKK-*α*, TRIF, Bcl-2, survivin	CDK2, CDK4	VEGF, VEGFR2	MyD88, TRAF6, IKK*α*, interferon *β*, ZEB1, ZEB2, snail, vimentin
